# Supplementary material for: Validation of a general subjective well-being factor using Classical Test Theory
Source: PeerJ. 2020 Jun 9;8:e9193. doi: 10.7717/peerj.9193 (PMC7292025; doi:10.7717/peerj.9193)
Supplement: Supplemental Information 2 [file peerj-08-9193-s002.pdf]

SUPPLEMENTARY MATERIAL

Validation of a General Subjective Well-Being Factor using Classical Test Theory

Ali Al Nima[1, 2]*, Kevin M. Cloninger[1, 3], Franco Lucchese[4], Sverker Sikström[5], Danilo Garcia[1, 2, 6]*

[1]Blekinge Center of Competence, Region Blekinge, Karlskrona, Sweden

[2]Department of Psychology, University of Gothenburg, Gothenburg, Sweden

[3]Anthropedia Foundation, St. Louis, Missouri, USA

[4]Department of Dynamic and Clinical Psychology, University of Rome "La Sapienza", Rome, Italy

[5]Depeartment of Psychology, Lund University, Lund, Sweden

[6]Department of Behavioral Sciences and Learning, Linköping University, Linköping, Sweden

*Correspondence concerning this article should be addressed to A. A. Nima, alinor_1979@yahoo.co.uk, and D. Garcia, danilo.garcia@icloud.com.

Table S1: Descriptive statistics for all the items and scales in the study (*N* = 527).

	Items	Range	Minimum	Maximum	Mean	*SD*	Skewness	Kurtosis
SWLS	In most ways my life is close to my ideal.	6.00	1.00	7.00	4.55	1.90	-0.60	-0.91
	The conditions of my life are excellent.	6.00	1.00	7.00	4.61	1.83	-0.64	-0.77
	I am satisfied with my life.	6.00	1.00	7.00	4.88	1.88	-0.86	-0.51
	So far I have gotten the important things I want in life.	6.00	1.00	7.00	4.89	1.82	-0.88	-0.41
	If I could live my life over. I would change almost nothing.	6.00	1.00	7.00	3.85	2.12	-0.01	-1.49
HILS	My lifestyle allows me to be in harmony.	6.00	1.00	7.00	4.86	1.68	-0.85	-0.21
	Most aspects of my life are in balance.	6.00	1.00	7.00	4.88	1.73	-0.87	-0.28
	I am in harmony.	6.00	1.00	7.00	4.78	1.76	-0.75	-0.48
	I accept the various conditions of my life.	6.00	1.00	7.00	5.37	1.43	-1.34	1.57
	I fit in well with my surroundings.	6.00	1.00	7.00	5.21	1.59	-1.10	0.56
NA	Distressed	4.00	1.00	5.00	1.91	1.11	1.18	0.50
	Upset	4.00	1.00	5.00	1.81	1.04	1.32	1.10
	Guilty	4.00	1.00	5.00	1.46	0.92	2.16	4.01
	Afraid	4.00	1.00	5.00	1.60	1.04	1.87	2.77
	Hostile	4.04	0.96	5.00	1.45	0.88	2.16	4.26
	Irritable	4.00	1.00	5.00	1.96	1.10	1.15	0.61
	Ashamed	4.00	1.00	5.00	1.44	0.91	2.35	5.26
	Nervous	4.00	1.00	5.00	1.92	1.16	1.18	0.41
	Jittery	4.00	1.00	5.00	1.65	1.05	1.68	2.01
	Scared	4.04	0.96	5.00	1.59	1.03	1.83	2.59
PA	Interested	4.00	1.00	5.00	3.56	0.99	-0.51	-0.15
	Enthusiastic	4.00	1.00	5.00	3.15	1.17	-0.21	-0.78
	Proud	4.00	1.00	5.00	3.01	1.30	-0.05	-1.10
	Alert	4.00	1.00	5.00	3.57	1.07	-0.56	-0.23
	Inspired	4.00	1.00	5.00	2.97	1.26	0.01	-1.03
	Determined	4.00	1.00	5.00	3.70	1.11	-0.62	-0.34
	Attentive	4.00	1.00	5.00	3.80	1.00	-0.84	0.58
	Active	4.00	1.00	5.00	3.27	1.17	-0.26	-0.73
	Excited	4.00	1.00	5.00	2.89	1.28	0.06	-1.06
	Strong	4.00	1.00	5.00	3.19	1.32	-0.22	-1.08
	**Scales**							
	SWBS	3.18	-2.07	1.11	0.00	0.66	-0.74	,06
	SWLS	6.00	1.00	7.00	4.56	1.72	-0.60	-.72
	HILS	6.00	1.00	7.00	5.02	1.50	-0.90	.10
	NA	4.00	1.00	5.00	1.68	0.83	1.56	2.06
	PA	4.00	1.00	5.00	3.31	0.90	-0.19	-.47

Note. SWBS = A simple average of standardized scores of the 30 items including raw average of the 10 Negative Affect (NA) reversed items, and raw averages of the five Satisfaction with Life Scale (SWLS) items, the five Harmony in Life Scale (HILS) items, and the 10 Positive Affect (PA) items.

Table S2. Map of correlations between the 30 items in the study (*N* = 527).

Table S2. Map of correlations (*N* =527).

	Items	1	2	3	4	5	6	7	8	9	10	11	12	13	14	15	16	17	18	19	20	21	22	23	24	25	26	27	28	29
SWLS	**In most ways my life is close to my ideal. (1)**																													
	**The conditions of my life are excellent. (2)**	.868**																												
	**I am satisfied with my life. (3)**	.857**	.825**																											
	**So far I have gotten the important things I want in life. (4)**	.819**	.756**	.839**																										
	**If I could live my life over, I would change almost nothing. (5)**	.701**	.663**	.675**	.673**																									
HILS	**My lifestyle allows me to be in harmony. (6)**	.777**	.739**	.792**	.715**	.580**																								
	**Most aspects of my life are in balance. (7)**	.787**	.761**	.811**	.727**	.577**	.889**																							
	**I am in harmony. (8)**	.773**	.719**	.797**	.719**	.591**	.903**	.911**																						
	**I accept the various conditions of my life. (9)**	.630**	.625**	.666**	.629**	.487**	.675**	.709**	.723**																					
	**I fit in well with my surroundings. (10)**	.703**	.684**	.727**	.680**	.538**	.794**	.818**	.811**	.767**																				
NA	**Distressed (11)**	-.478**	-.490**	-.520**	-.418**	-.324**	-.521**	-.550**	-.576**	-.513**	-.489**																			
	**Upset (12)**	-.518**	-.522**	-.552**	-.452**	-.363**	-.552**	-.575**	-.590**	-.518**	-.495**	.835**																		
	**Guilty (13)**	-.294**	-.283**	-.323**	-.318**	-.166**	-.333**	-.376**	-.367**	-.362**	-.304**	.526**	.609**																	
	**Afraid (14)**	-.399**	-.401**	-.446**	-.371**	-.245**	-.412**	-.464**	-.433**	-.421**	-.427**	.673**	.713**	.624**																
	**Hostile (15)**	-.322**	-.328**	-.379**	-.358**	-.224**	-.397**	-.410**	-.425**	-.409**	-.367**	.592**	.608**	.524**	.579**															
	**Irritable (16)**	-.427**	-.392**	-.447**	-.408**	-.286**	-.457**	-.465**	-.447**	-.462**	-.447**	.677**	.701**	.523**	.567**	.655**														
	**Ashamed (17)**	-.296**	-.288**	-.344**	-.320**	-.174**	-.352**	-.388**	-.392**	-.403**	-.361**	.531**	.569**	.731**	.629**	.521**	.516**													
	**Nervous (18)**	-.425**	-.407**	-.436**	-.360**	-.280**	-.470**	-.491**	-.498**	-.412**	-.437**	.707**	.692**	.559**	.677**	.559**	.624**	.547**												
	**Jittery (19)**	-.366**	-.351**	-.378**	-.371**	-.250**	-.408**	-.444**	-.436**	-.405**	-.387**	.573**	.624**	.541**	.580**	.564**	.568**	.520**	.640**											
	**Scared (20)**	-.409**	-.395**	-.456**	-.385**	-.262**	-.432**	-.462**	-.447**	-.433**	-.427**	.682**	.734**	.558**	.841**	.570**	.551**	.628**	.676**	.590**										
PA	**Interested (21)**	.506**	.460**	.501**	.450**	.351**	.520**	.484**	.530**	.530**	.525**	-.385**	-.391**	-.294**	-.326**	-.349**	-.398**	-.322**	-.346**	-.282**	-.340**									
	**Enthusiastic (22)**	.463**	.452**	.477**	.455**	.373**	.456**	.428**	.482**	.485**	.510**	-.358**	-.325**	-.167**	-.239**	-.240**	-.325**	-.188**	-.248**	-.226**	-.268**	.667**								
	**Proud (23)**	.504**	.442**	.508**	.453**	.436**	.429**	.439**	.456**	.431**	.478**	-.300**	-.341**	-.207**	-.249**	-.211**	-.286**	-.237**	-.220**	-.185**	-.289**	.562**	.687**							
	**Alert (24)**	.213**	.172**	.197**	.199**	.117**	.202**	.207**	.233**	.263**	.218**	-.192**	-.145**	-.116**	-.160**	-.165**	-.164**	-.116**	-.153**	-.085	-.169**	.487**	.389**	.308**						
	**Inspired (25)**	.380**	.311**	.362**	.329**	.283**	.367**	.319**	.372**	.325**	.362**	-.217**	-.219**	-.036	-.129**	-.122**	-.228**	.057	-.153**	-.135**	-.171**	.580**	.684**	.618**	.314**					
	**Determined (26)**	.285**	.255**	.274**	.283**	.209**	.306**	.309**	.332**	.342**	.362**	-.222**	-.173**	-.146**	-.169**	-.210**	-.202**	-.180**	-.163**	-.143**	-.177**	.566**	.537**	.510**	.434**	.526**				
	**Attentive (27)**	.286**	.256**	.291**	.292**	.181**	.300**	.315**	.330**	.386**	.350**	-.255**	-.218**	-.151**	-.213**	-.255**	-.268**	-.177**	-.182**	-.162**	-.201**	.621**	.487**	.392**	.630**	.413**	.635**			
	**Active (28)**	.345**	.306**	.317**	.307**	.228**	.336**	.334**	.370**	.332**	.350**	-.245**	-.220**	-.117**	-.152**	-.145**	-.245**	-.136**	-.184**	-.103*	-.205	.495**	.566**	.500**	.514**	.514**	.525**	.566**		
	**Excited (29)**	.427**	.390**	.432**	.379**	.366**	.362**	.334**	.387**	.386**	.406**	-.232**	-.203**	-.083	-.101*	-.126**	-.239**	-.097*	-.139**	-.076	-.140**	.540**	.742**	.699**	.270**	.662**	.461**	.392**	.531**	
	**Strong (30)**	.462**	.453**	.494**	.435**	.403**	.444**	.426**	.483**	.452**	.482**	-.322**	-.332**	-.179**	-.232**	-.222**	-.333**	-.231**	-.254**	-.224**	-.257**	.549**	.643**	.682**	.399**	.618**	.583**	.490**	.623**	.625**

Note: Higher correlations are darker gray and lower correlations are lighter gray, ** p < .01, blue = * p <.05, green = *ns*. SWLS = Satisfaction with Life Scale; HILS = Harmony in Life Scale; NA = Negative Affect; PA = Positive Affect.

Table S3. Correlations and Cronbach's alphas for SWBS and its four subscales (*N* = 527).

Scales	1	2	3	4	5
SWLS average of raw scores (1)	**.94**				
HILS average of raw scores (2)	.83**	**.95**			
NA average of raw scores (3)	-.51**	-.60**	**.94**		
PA average of raw scores (4)	.52**	.55**	-.34**	**.92**	
SWBS (5)	.82**	.87**	-.79**	.77**	**.96**

Note: ** $p < .01$, SWBS = A simple average of standardized scores of the 30 items including raw average of the 10 Negative Affect (NA) reversed items, and raw averages of the five Satisfaction with Life Scale (SWLS) items, the five Harmony in Life Scale (HILS) items, and the 10 Positive Affect (PA) items. Numbers in diagonal and boldfaced refer to the *Cronbach's alphas*.

Table S4: Standardized loadings factor of bifactor confirmatory factor analysis for the general latent trait and its specific latent traits (*N* =527).

	Items	SWBS	SWLS	HILS	PA	NA	h2	u2	p2
	**In most ways my life is close to my ideal.**	0.82	0.47				0.89	0.11	0.75
	**The conditions of my life are excellent.**	0.78	0.45				0.81	0.19	0.75
**SWLS**	**I am satisfied with my life.**	0.85	0.37				0.86	0.14	0.84
	**So far I have gotten the important things I want in life.**	0.77	0.42				0.77	0.23	0.77
	**If I could live my life over. I would change almost nothing.**	0.62	0.42				0.56	0.44	0.69
	**My lifestyle allows me to be in harmony.**	0.92		0.18			0.88	0.12	0.96
	**Most aspects of my life are in balance.**	0.94		0.13			0.90	0.10	0.98
**HILS**	**I am in harmony.**	0.96		0.13			0.94	0.06	0.98
	**I accept the various conditions of my life.**	0.82		0.46			0.88	0.12	0.76
	**I fit in well with my surroundings.**	0.88		0.11			0.79	0.21	0.98
	**Interested**	0.57			0.52		0.60	0.40	0.55
	**Enthusiastic**	0.52			0.67		0.72	0.28	0.38
	**Proud**	0.50			0.61		0.62	0.38	0.40
	**Alert**	0.25			0.45		0.27	0.74	0.24
**PA**	**Inspired**	0.39			0.67		0.60	0.40	0.25
	**Determined**	0.36			0.60		0.49	0.51	0.26
	**Attentive**	0.37			0.53		0.42	0.58	0.33
	**Active**	0.38			0.60		0.50	0.50	0.29
	**Excited**	0.42			0.67		0.63	0.37	0.28
	**Strong**	0.51			0.62		0.64	0.36	0.40
	**Distressed**	0.60				-0.60	0.72	0.28	0.50
	**Upset**	0.62				-0.63	0.78	0.22	0.49
	**Guilty**	0.39				-0.60	0.51	0.49	0.30
	**Afraid**	0.48				-0.71	0.73	0.27	0.31
	**Hostile**	0.45				-0.55	0.51	0.50	0.40
**NA**	**Irritable**	0.52				-0.54	0.56	0.44	0.48
	**Ashamed**	0.42				-0.58	0.51	0.49	0.34
	**Nervous**	0.51				-0.62	0.64	0.36	0.40
	**Jittery**	0.46				-0.55	0.51	0.49	0.41
	**Scared**	0.49				-0.70	0.73	0.27	0.33
	**Omega-total (ω*Total*)**	0.79							
	**Omega Hierarchical (ω*H*)**	0.63							
	**Omega subscale (ω*S*)**		0.94	0.98	0.72	0.81			
	**Omega hirerarchial subscale (ω*HS*)**		0.22	0.05	0.48	0.49			
	**Omega general for subscale**		0,72	0,93	0,25	0,32			
	**ECV**	0.57							
	**Eigenvalues**	11.47	0.91	0.29	3.58	3.73			

Note. h2 = communalities, u2 = error variance (uniqueness), p2 = item explained common variance (I-ECV), Raw items of Negative Affect (NA) are reversed. Absolut values of negative loadings of two Harmony in Life Scale (HILS) items (HILS4 and HILS5) are used. SWLS = Satisfaction with Life Scale; PA = Positive Affect.

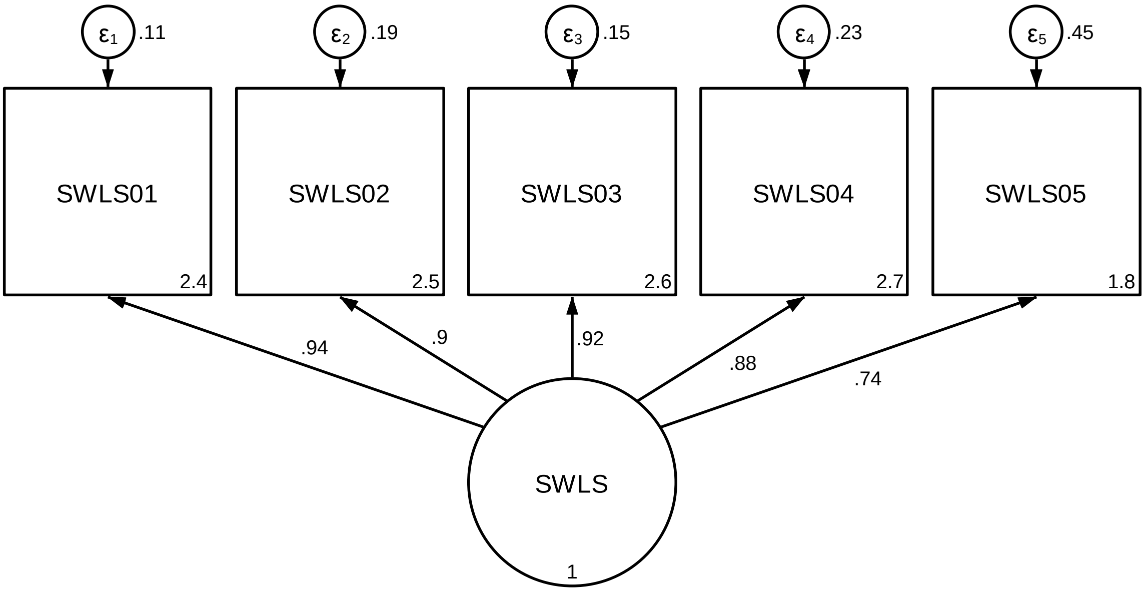

Figure S1. Structural equation model of Satisfaction with Life (SWLS). Showing all paths (from SWLS to its items) and their standardized parameter estimates.

Note: Chi-square value (χ_2 = 45.30, *df* = 5, *p* < .001), Satorra Bentler χ_2 (S-B χ_2 = 26.38, *df* = 5, *p* < .001), CFI = .99, TLI = .98 and RMSEA = .09. RMSEA, CFI, and TLI goodness-of-fit statistics were computed using the Satorra–Bentler scaled chi-squared statistic (*N* =527).

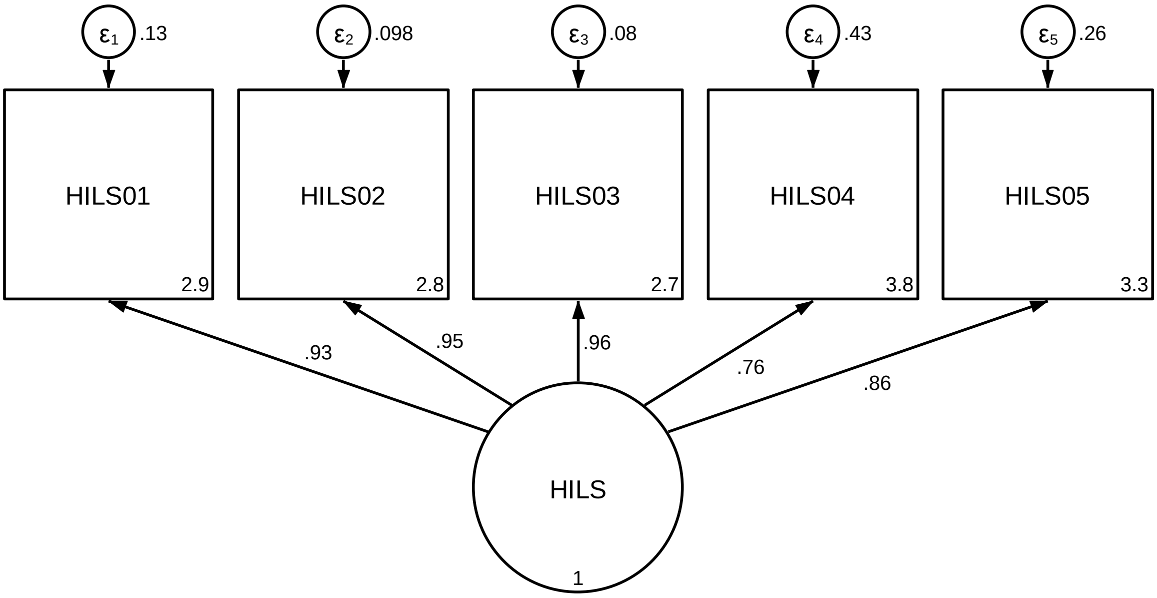

Figure S2. Structural equation model of Harmony in Life Scale (HILS). Showing all paths (from HILS to its items) and their standardized parameter estimates.

Note: Chi-square value (χ_2 = 87.65, *df* = 5, *p* < .001), Satorra Bentler χ_2 (S-B χ_2 = 47.41, *df* = 5, *p* < .001), CFI = .98, TLI = .96 and RMSEA = .13. RMSEA, CFI, and TLI goodness-of-fit statistics were computed using the Satorra–Bentler scaled chi-squared statistic (*N* =527).

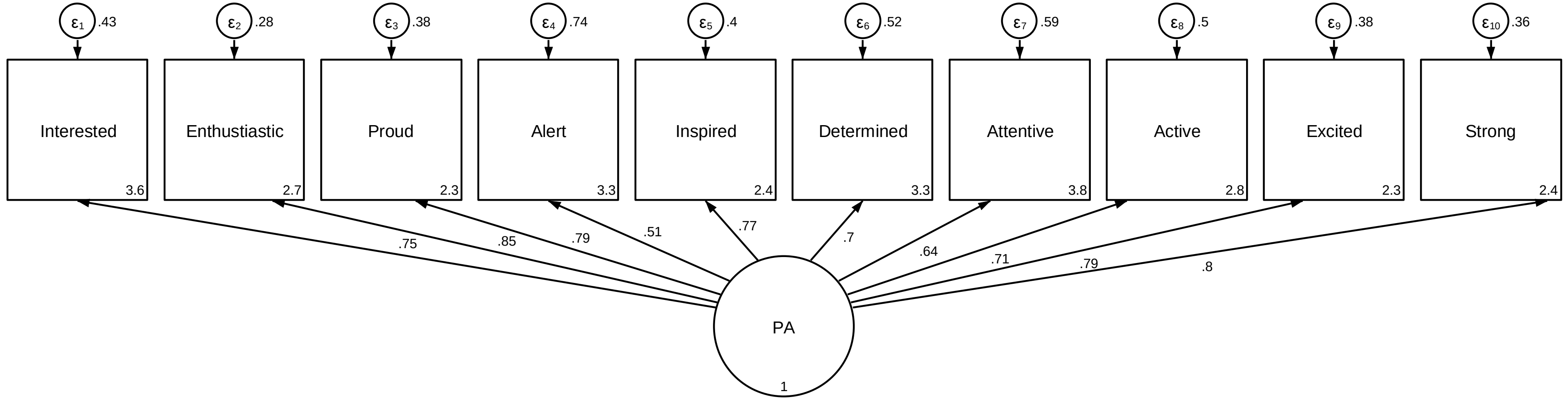

Figure S3. Structural equation model of Positive Affect (PA). Showing all paths (from PA to its items) and their standardized parameter estimates.

Note: Chi-square value (χ_2 = 508.33, *df* = 35, *p* < .001), Satorra Bentler χ_2 (S-B χ_2 = 350.72, *df* = 35, *p* < .001), CFI = .88, TLI = .84 and RMSEA = .13. RMSEA, CFI, and TLI goodness-of-fit statistics were computed using the Satorra–Bentler scaled chi-squared statistic (*N* =527).

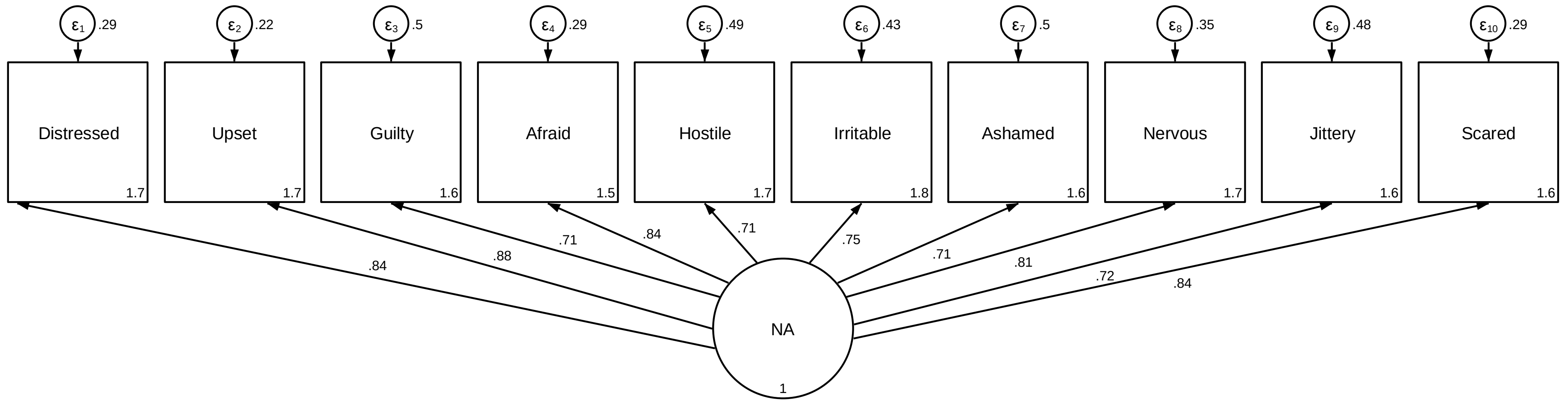

Figure S4. Structural equation model of Negative Affect (NA). Showing all paths (from NA to its items) and their standardized parameter estimates.

Note: Chi-square value ($\chi2$ = 520.12, *df* = 35, *p* < .001), Satorra Bentler $\chi2$ (S-B $\chi2$ = 233.83, *df* = 35, *p* < .001), CFI = .90, TLI = .89 and RMSEA = .10. RMSEA, CFI, and TLI goodness-of-fit statistics were computed using the Satorra–Bentler scaled chi-squared statistic (*N* =527).

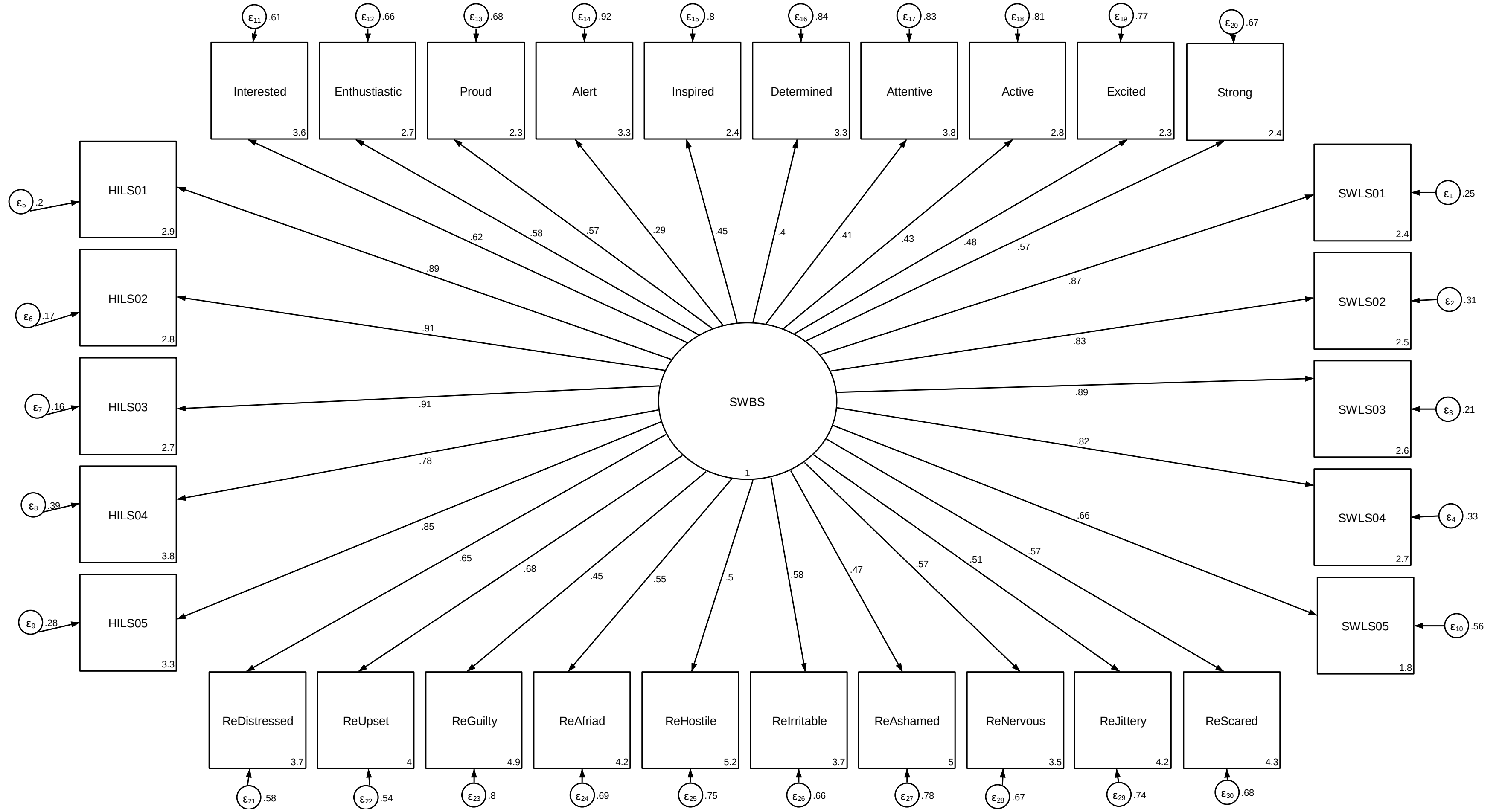

Figure S5. Structural equation model of Subjective Well-Being (SWBS). Showing all paths (from SWBS to its items) and their standardized parameter estimates.

Note: Chi-square value (χ_2 = 6613.79, *df* = 405, *p* < .001), Satorra Bentler χ_2 (*S-B* χ_2 = 4991.18, *df* = 405, *p* < .001), CFI = .58, TLI = .55 and RMSEA = .15. RMSEA, CFI, and TLI goodness-of-fit statistics were computed using the Satorra–Bentler scaled chi-squared statistic. All items for Negative Affect are reversed (*N* =527).

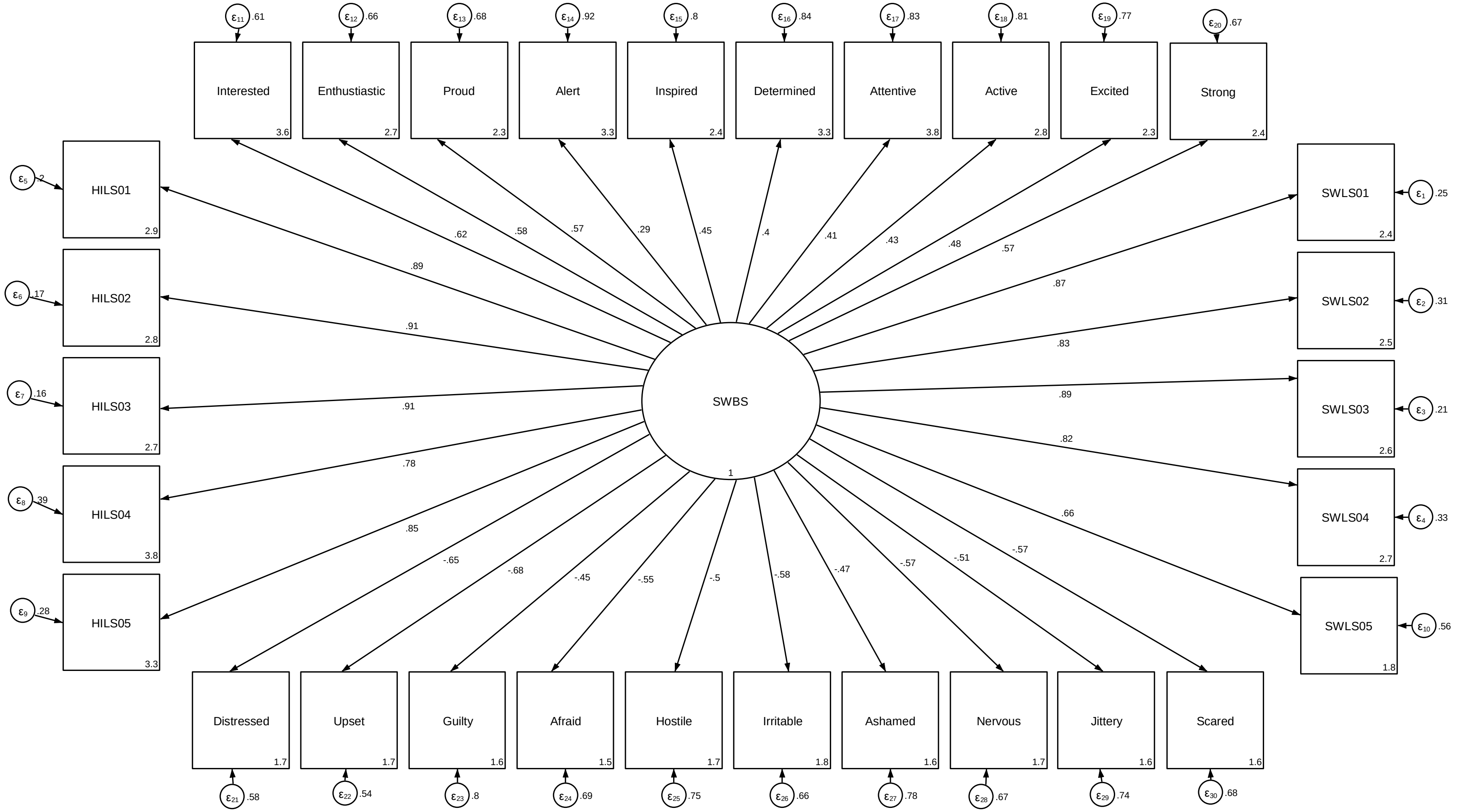

Figure S6. Structural equation model of Subjective Well-Being (SWBS). Showing all paths (from SWBS to its items) and their standardized parameter estimates.

Note: Chi-square value ($\chi 2$ = 6613.79, *df* = 405, *p* < .001), Satorra Bentler $\chi 2$ (*S-B* $\chi 2$ = 4991.18, *df* = 405, *p* < .001), CFI = .58, TLI = .55 and RMSEA = .15. RMSEA, CFI, and TLI goodness-of-fit statistics were computed using the Satorra–Bentler scaled chi-squared statistic (*N* =527).

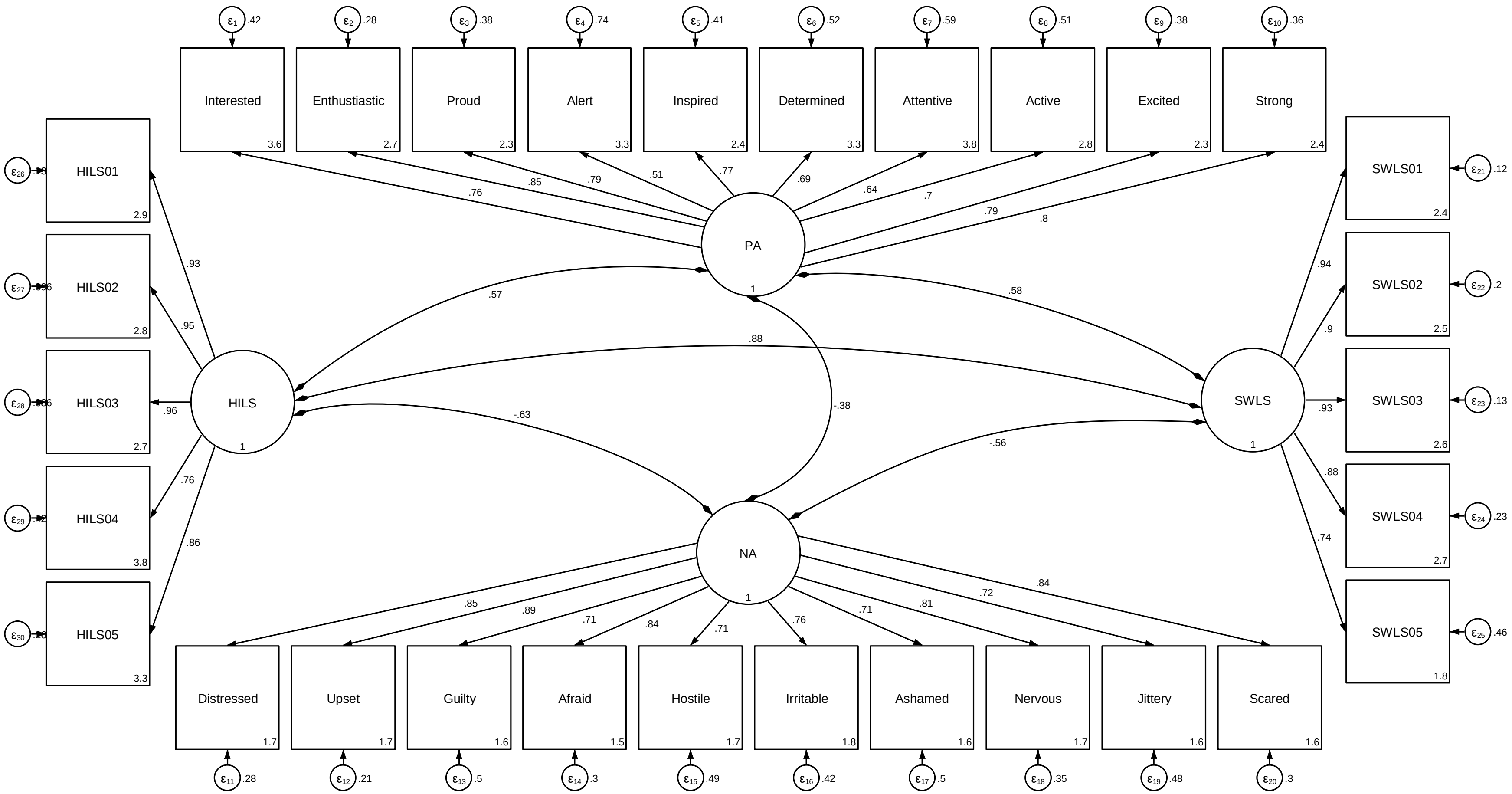

Figure S7. Structural equation model of the multidimensional correlated model for the Subjective Well-Being subscales. Showing all correlations between subscales and all paths (from subscales to their items) and their standardized parameter estimates.

Note: Chi-square value (χ_2 = 1875.24, *df* = 399, *p* < .001), Satorra Bentler χ_2 (*S-B* χ_2 = 1413.64, *df* = 399, *p* < .001), CFI=.91, TLI = .90 and RMSEA = .07. RMSEA, CFI, and TLI goodness-of-fit statistics were computed using the Satorra–Bentler scaled chi-squared statistic (*N* =527). SWLS = Satisfaction with Life Scale; HILS = Harmony in Life Scale; NA = Negative Affect; PA = Positive Affect.

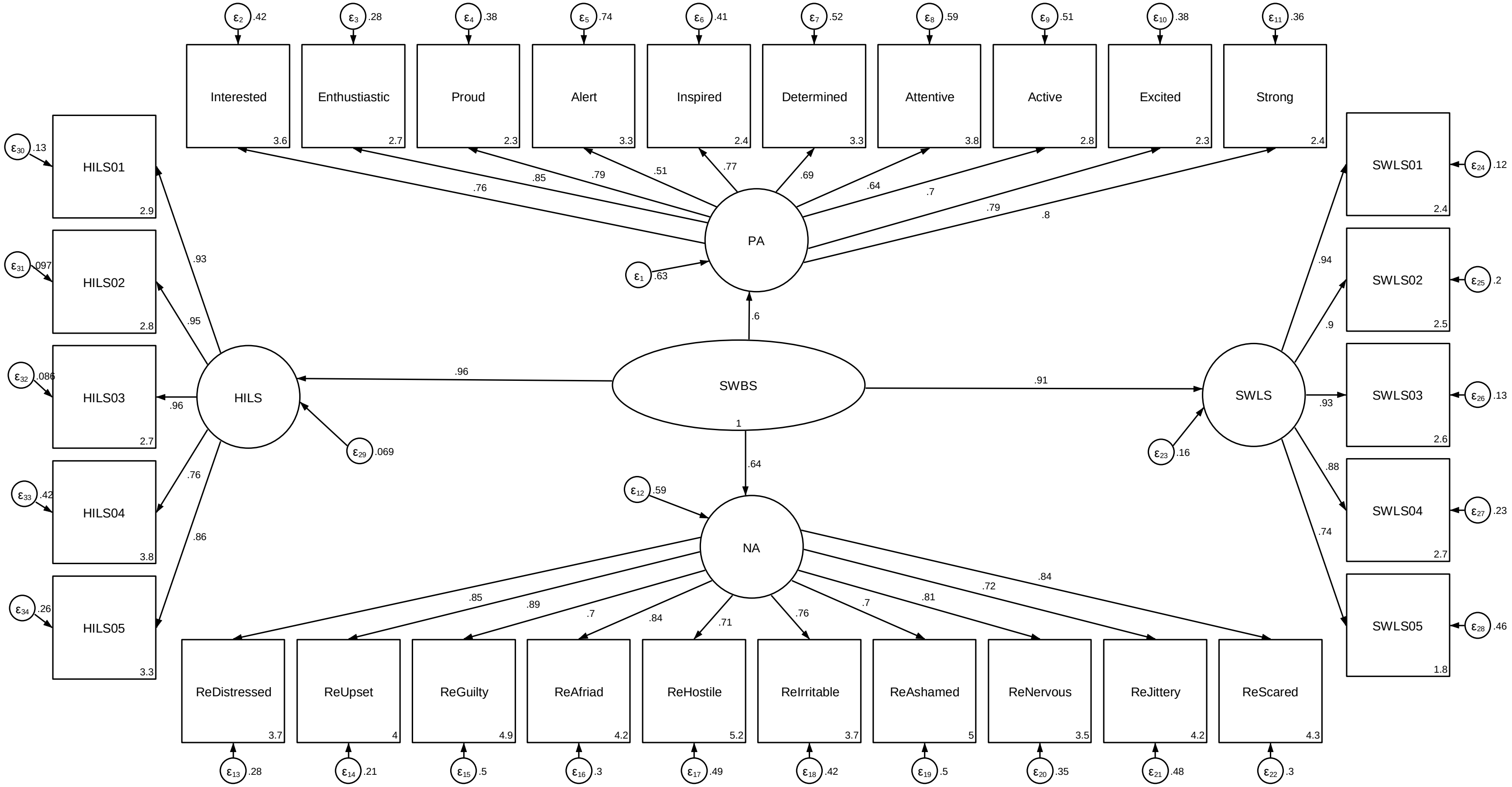

Figure S8. Structural equation model of higher order factor model (second order model) of Subjective Well-Being (SWBS) and its specific subscales. Showing all paths (from SWBS to specific subscales, and from specific subscales to their items) and their standardized parameter estimates.

Note: Chi-square value (χ_2 = 1881.49, *df* = 401, *p* < .001), Satorra Bentler χ_2 (*S-B* χ_2 = 1419.90, *df* = 401, *p* < .001), CFI = .91, TLI = .90 and RMSEA = .07. RMSEA, CFI, and TLI goodness-of-fit statistics were computed using the Satorra–Bentler scaled chi-squared statistic (*N* =527). SWLS = Satisfaction with Life Scale; HILS = Harmony in Life Scale; NA = Negative Affect (reversed items); PA = Positive Affect.

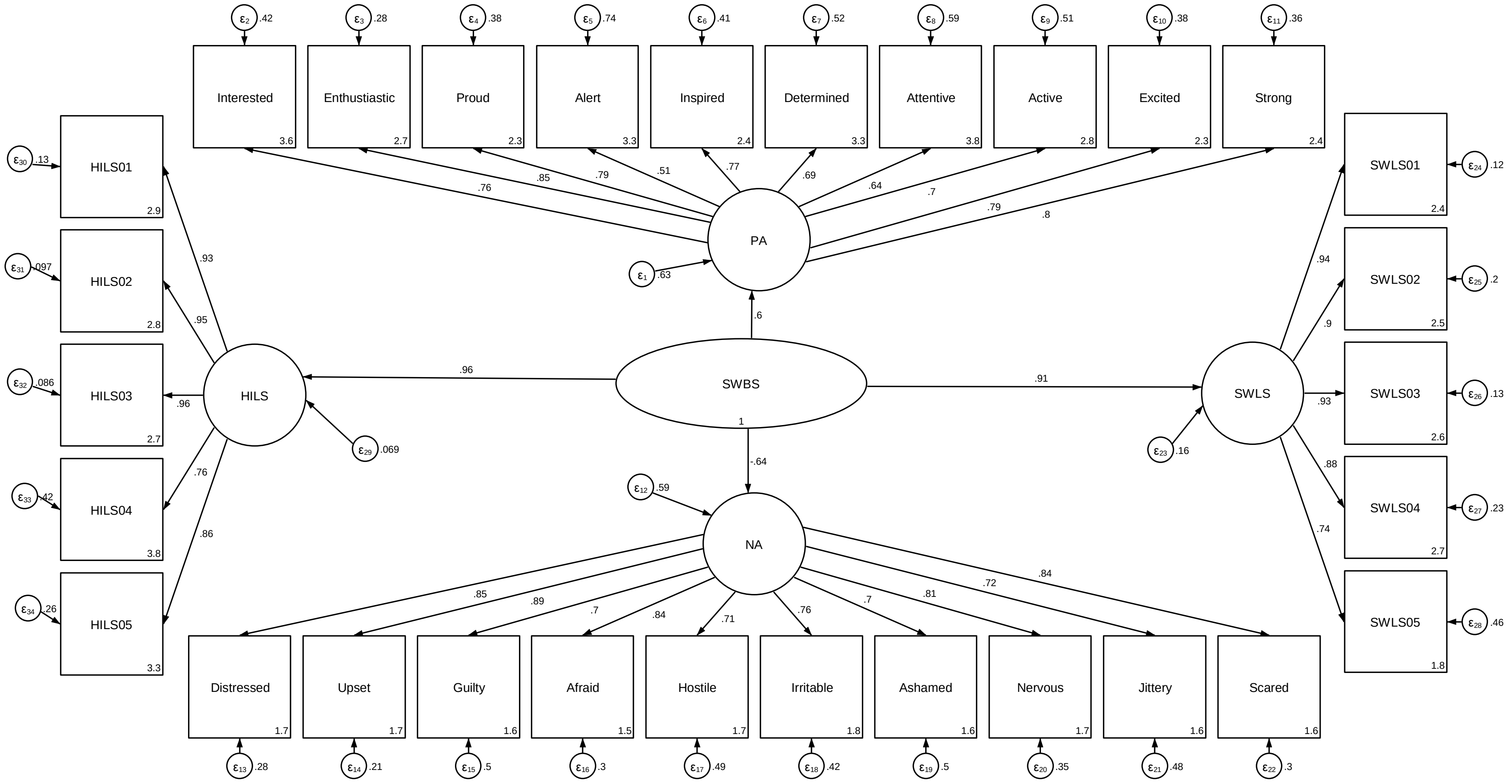

Figure S9. Structural equation model of higher order factor model (second order model) of Subjective Well-Being (SWBS) and its specific subscales. Showing all paths (from SWBS to specific subscales, and from specific subscales to their items) and their standardized parameter estimates.

Note: Chi-square value (χ_2 = 1881.49, *df* = 401, *p* < .001), Satorra Bentler χ_2 (*S-B* χ_2 = 1419.90, *df* = 401, *p* < .001), CFI = .91, TLI = .90 and RMSEA = .07. RMSEA, CFI, and TLI goodness-of-fit statistics were computed using the Satorra–Bentler scaled chi-squared statistic (*N* =527). SWLS = Satisfaction with Life Scale; HILS = Harmony in Life Scale; NA = Negative Affect; PA = Positive Affect.

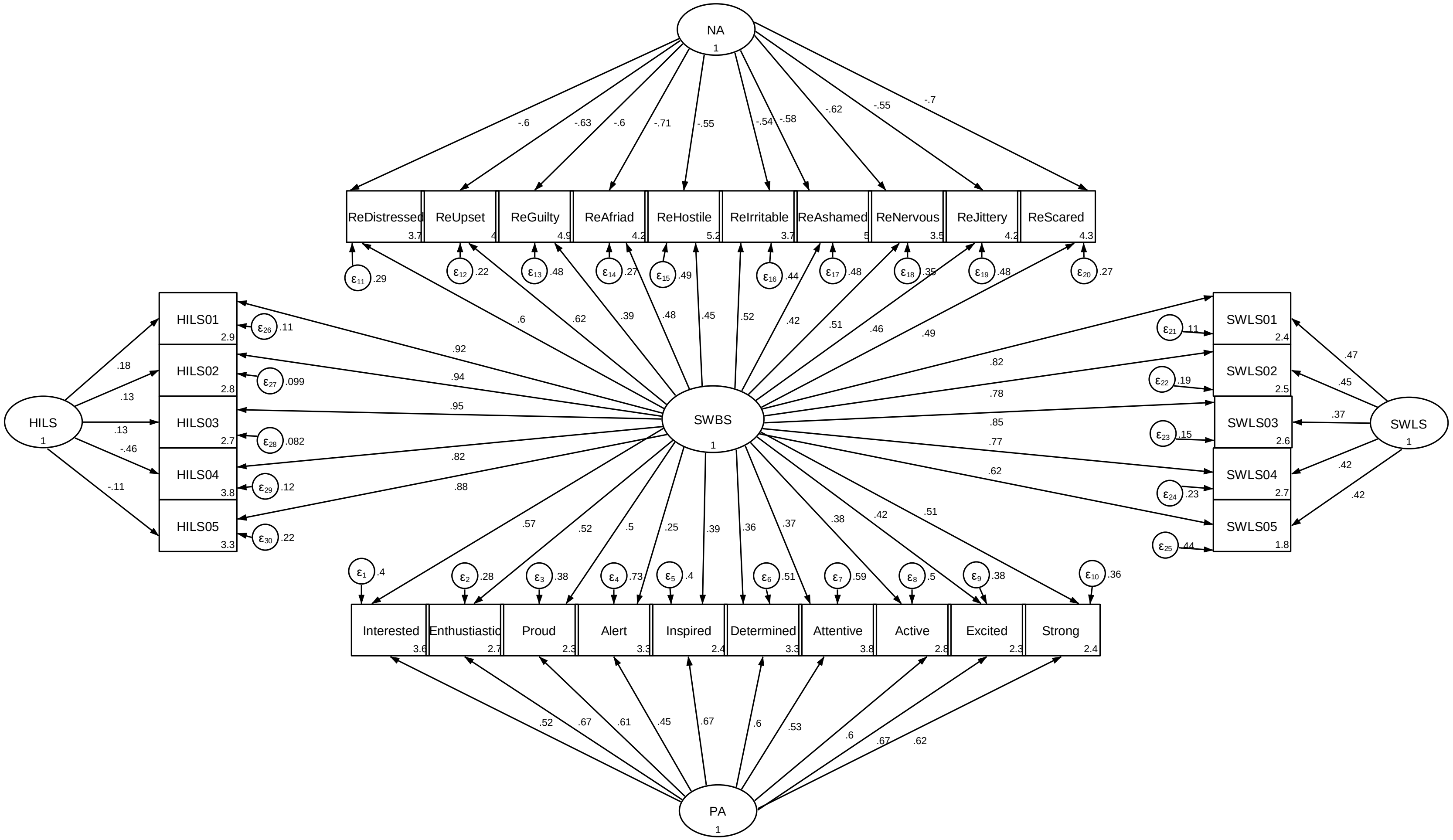

Figure S10. Structural equation model of bifactor model of Subjective Well-Being (SWBS) and its specific subscales. All paths (from SWBS to each item, and from specific subscales to their items) and their standardized parameter estimates.

Notes: Chi-square value (χ_2 = 1660.78, *df* = 375, *p* < .001), Satorra Bentler χ_2 (*S-B* χ_2 =1265.80, *df* = 375, *p* < .001), CFI = .92, TLI = .91 and RMSEA = .067. RMSEA, CFI, and TLI goodness-of-fit statistics were computed using the Satorra–Bentler scaled chi-squared statistic (*N* =527). SWLS = Satisfaction with Life Scale; HILS = Harmony in Life Scale; NA = Negative Affect (reversed items); PA = Positive Affect.

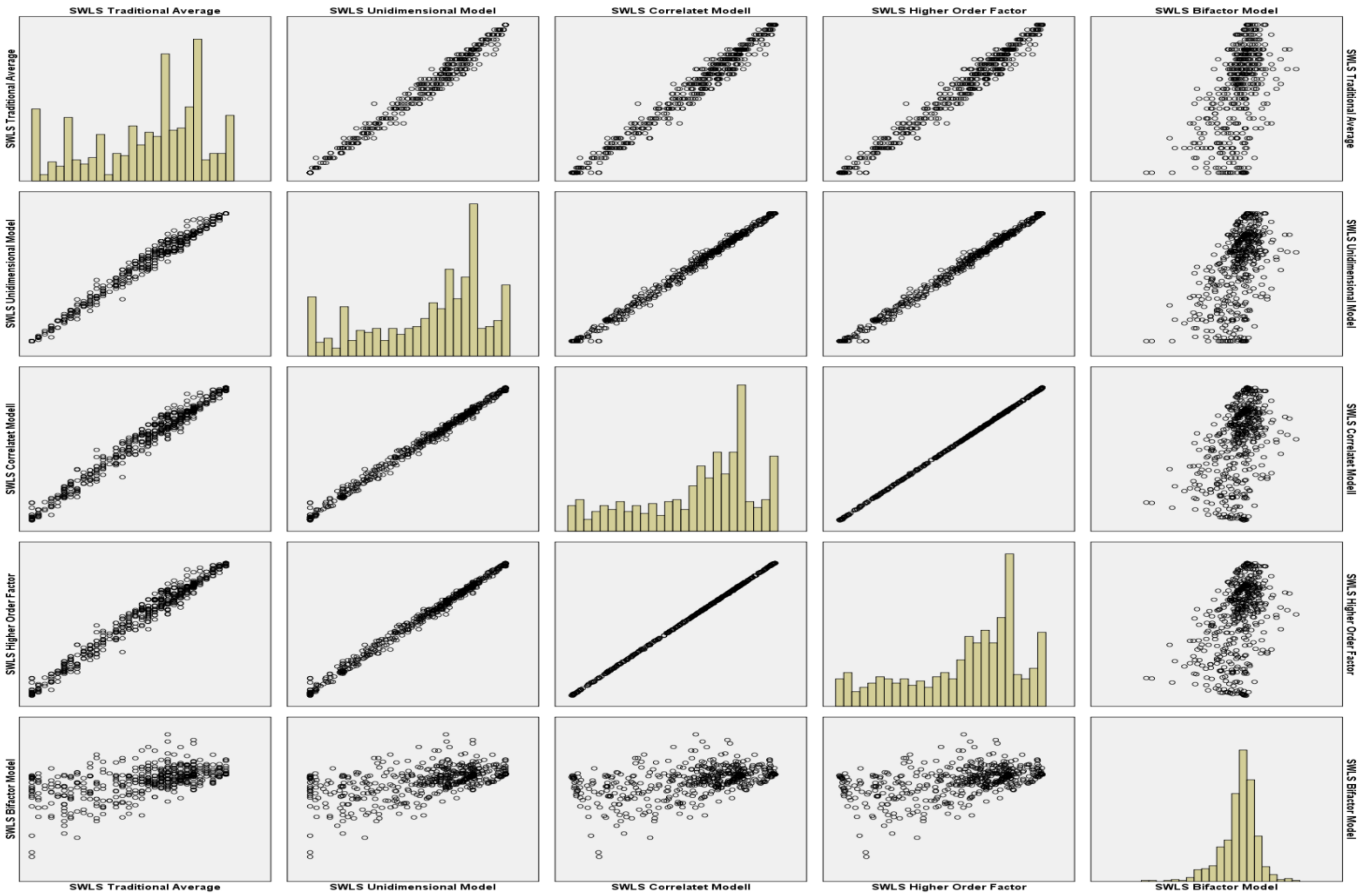

Figure S11. Scatterplot matrix of correlations and distributions of scores for Satisfaction with Life Scale (SWLS) across the different models in the present study (i.e., traditional average score approach, unidimensional model, correlated factors model, second order factor model and bifactor model). Reversed items of the Negative Affect (NA) are used when second order factor model and bifactor model are applied (*N* = 527).

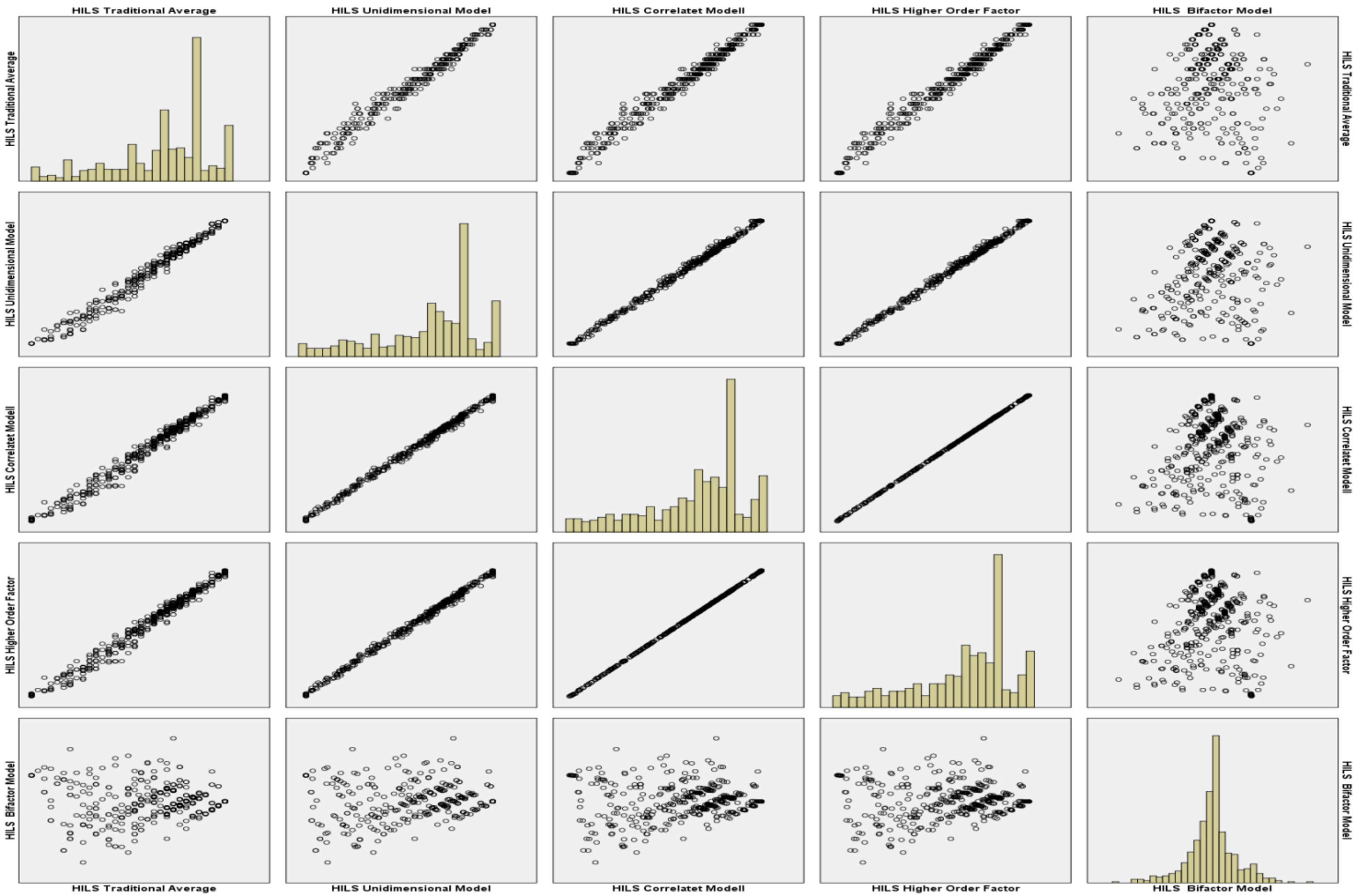

Figure S12. Scatterplot matrix of correlations and distributions of scores for Harmony in Life (HILS) across the different models in the present study (i.e., traditional average score approach, unidimensional model, correlated factors model, second order factor model and bifactor model). Reversed items of the Negative Affect (NA) are used when second order factor model and bifactor model are applied (*N* = 527).

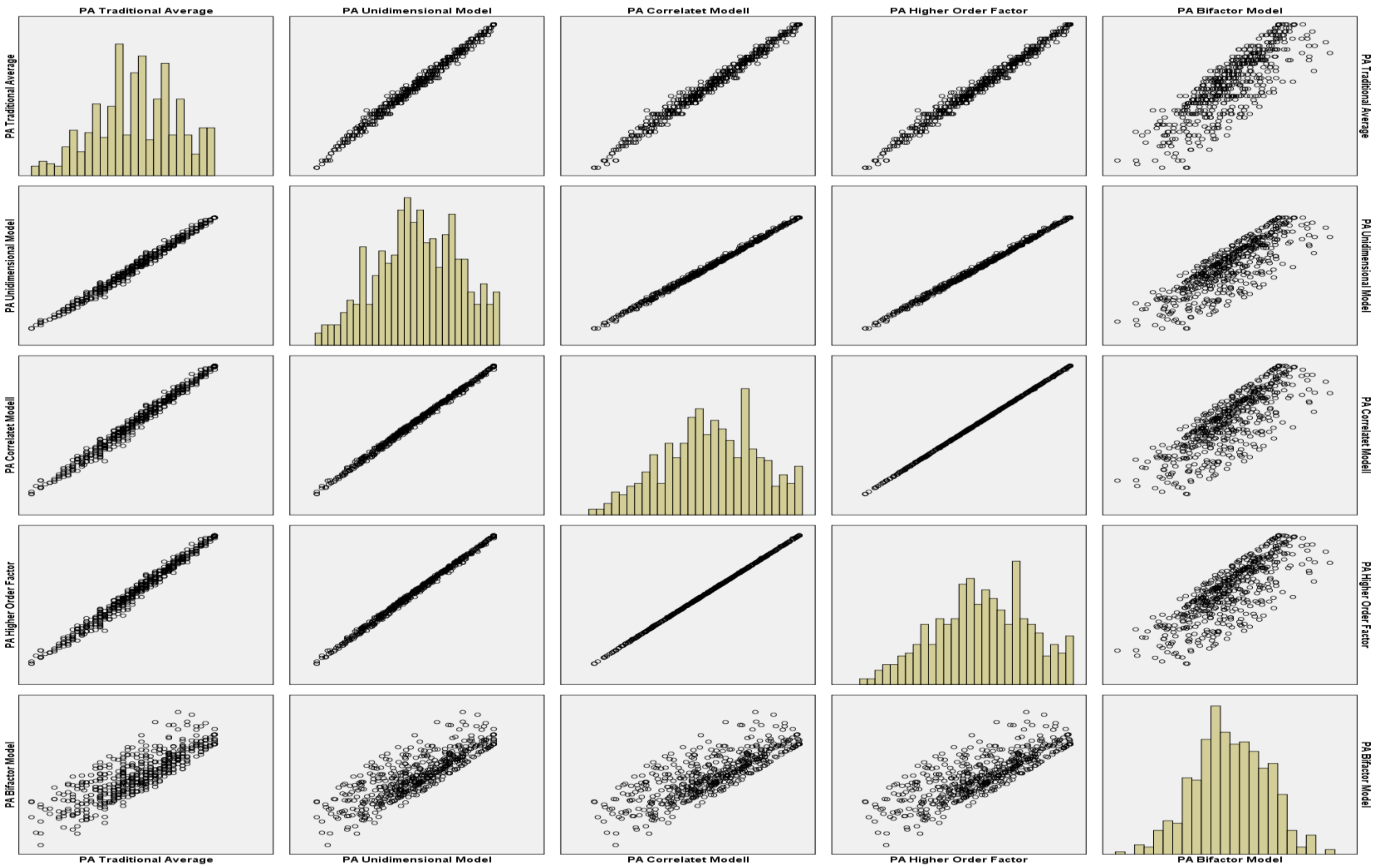

Figure S13. Scatterplot matrix of correlations and distributions of scores for Positive Affect (PA) across the different models in the present study (i.e., traditional average score approach, unidimensional model, correlated factors model, second order factor model and bifactor model). Reversed items of the Negative Affect (NA) are used when second order factor model and bifactor model are applied (N = 527).

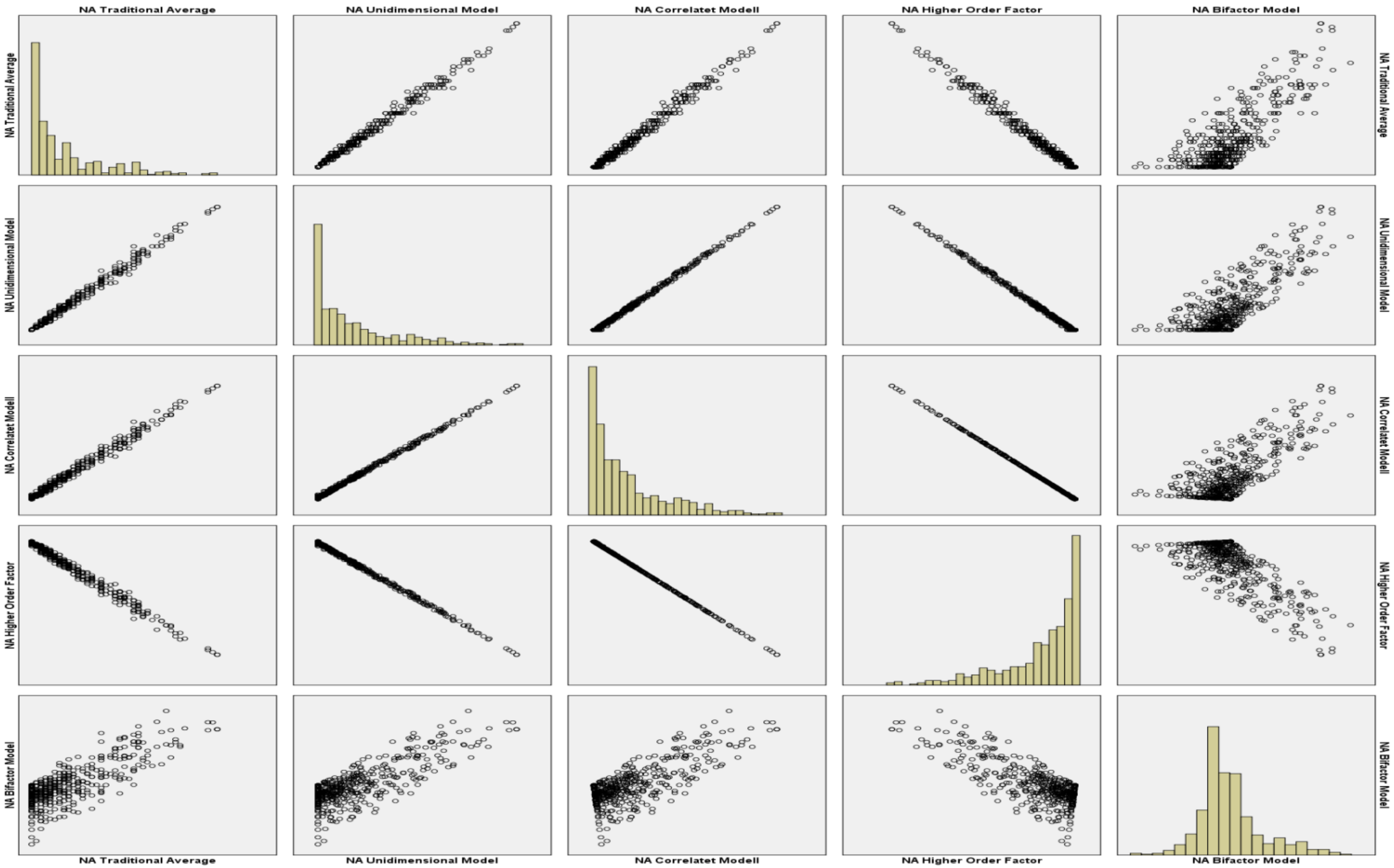

Figure S14. Scatterplot matrix of correlations and distributions of scorers for Negative Affect across the different models in the present study (i.e., traditional average score approach, unidimensional model, correlated factors model, second order factor model and bifactor model). Reversed items of the Negative Affect (NA) are used when second order factor model and bifactor model are applied (N = 527).

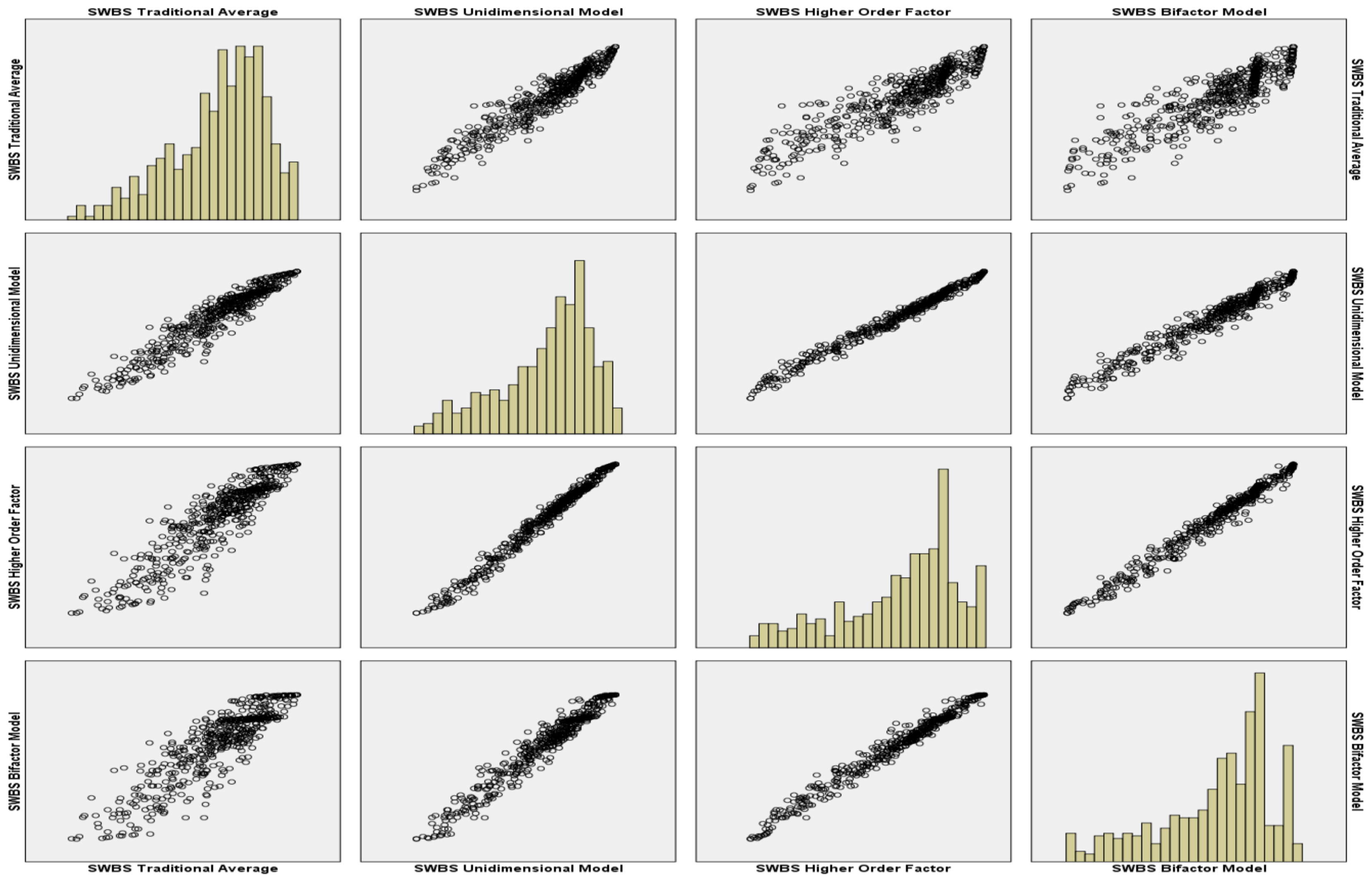

Figure S15. Scatterplot matrix of correlations and distributions of scores for Subjective Well-Being (SWBS) across different models (traditional average score approach, unidimensional model, second order factor model and bifactor model), Reversed items of the NA are used when traditional average score approach, unidimensional second order factor model and bifactor model are applied (N = 527).
